# Effects of Canonical NF-**κ**B Signaling Pathway on the Proliferation and Odonto/Osteogenic Differentiation of Human Stem Cells from Apical Papilla

**DOI:** 10.1155/2014/319651

**Published:** 2014-04-23

**Authors:** Junjun Li, Ming Yan, Zilu Wang, Shuanglin Jing, Yao Li, Genxia Liu, Jinhua Yu, Zhipeng Fan

**Affiliations:** ^1^Institute of Stomatology, Nanjing Medical University, 140 Hanzhong Road, Nanjing, Jiangsu 210029, China; ^2^Endodontic Department, School of Stomatology, Nanjing Medical University, 136 Hanzhong Road, Nanjing, Jiangsu 210029, China; ^3^Laboratory of Molecular Signaling and Stem Cells Therapy and Molecular Laboratory for Gene Therapy and Tooth Regeneration, Beijing Key Laboratory of Tooth Regeneration and Function Reconstruction, School of Stomatology, Capital Medical University, 4 Tian Tan Xi Li, Beijing 100050, China

## Abstract

*Background Information*. NF-**κ**B signaling pathway plays a complicated role in the biological functions of mesenchymal stem cells. However, the effects of NF-**κ**B pathway on the odonto/osteogenic differentiation of stem cells from apical papilla (SCAPs) remain unclear. The present study was designed to evaluate the effects of canonical NF-**κ**B pathway on the osteo/odontogenic capacity of SCAPs *in vitro*. *Results*. Western blot results demonstrated that NF-**κ**B pathway in SCAPs was successfully activated by TNF-**α** or blocked by BMS-345541. NF-**κ**B pathway-activated SCAPs presented a higher proliferation activity compared with control groups, as indicated by dimethyl-thiazol-diphenyl tetrazolium bromide assay (MTT) and flow cytometry assay (FCM). Wound scratch assay revealed that NF-**κ**B pathway-activated SCAPs presented an improved migration capacity, enhanced alkaline phosphatase (ALP) activity, and upregulated mineralization capacity of SCAPs, as compared with control groups. Meanwhile, the odonto/osteogenic markers (*ALP*/ALP, *RUNX2*/RUNX2, *OSX*/OSX, *OCN*/OCN, *OPN*/OPN, *BSP*/BSP, *DSPP*/DSP, and *DMP-1*/DMP-1) in NF-**κ**B pathway-activated SCAPs were also significantly upregulated as compared with control groups at both protein and mRNA levels. However, NF-**κ**B pathway-inhibited SCAPs exhibited a lower proliferation/migration capacity, and decreased odonto/osteogenic ability in comparison with control groups. *Conclusion*. Our findings suggest that classical NF-**κ**B pathway plays a paramount role in the proliferation and committed differentiation of SCAPs.

## 1. Introduction

NF-*κ*B pathway regulates the expression of a multitude of genes involved in the immune system, growth, inflammation, and cancer development [1–3]. It is commonly believed that NF-*κ*B pathway plays an important role during the tooth organogenesis and eruption process [4]. Furthermore, NF-*κ*B pathway interacts with other signaling pathways such as Notch signaling and PI3 K/Akt pathway during the tooth development and inflammation [5, 6]. The abolition of NF-*κ*B pathway may result in a developmental arrest of teeth [7]. However, the influence of NF-*κ*B pathway on tooth development as well as root maturation has not been fully clarified.

Tooth root development is an independent process after the formation of tooth crown, during which stem cells from apical papilla (SCAPs) are believed to play a crucial role [8]. SCAPs were a unique population of stem cells gathered in the immature root apex with the capacity of self-renewal and multiple differentiation [9]. Compared with dental pulp stem cells (DPSCs), SCAPs take on a higher proliferative ability and stronger multipotent differentiation potential. Furthermore, the strong expression of CD24 (pluripotency marker) and DSPP supports the concept that SCAPs are the best candidate for tooth regeneration and rehabilitation of damage [9–11]. Nevertheless, there is no convincing scientific evidence about the effects of NF-*κ*B pathway on the proliferation and differentiation of SCAPs.

NF-*κ*B pathway can be triggered in dental pulp stem cells under various stimulating factors, that is, trauma, inflammatory factors, MTA, and estrogen [12–15], in which tumor necrosis factor-*α* (TNF-*α*) is the putative classical pathway activator [4, 16, 17]. Meanwhile, this pathway can be effectively inhibited by 4(2′-aminoethyl) amino-1,8-dimethylimidazo(1,2-a) quinoxaline (BMS-345541, a selective inhibitor of IKK) [18, 19]. In this study, we hypothesize that activation or inhibition of NF-*κ*B pathway can affect the pace of proliferation and differentiation in SCAPs. To test this hypothesis, SCAPs were isolated from the developing apical papillae, and 10 ng/mL TNF-*α* or 1 *μ*mol/L BMS-345541 was, respectively, used to activate or inhibit NF-*κ*B pathway in SCAPs [15, 20]. The present findings revealed that the proliferative ability, migration potential, and odonto/osteogenic differentiation potential of SCAPs can be significantly affected by the activation or inhibition of NF-*κ*B pathway. These results provide novel insights into the role of classical NF-*κ*B pathway during the modification of mesenchymal stem cells and stem cell-based tooth regeneration.

## 2. Materials and Methods

### 2.1. Cell Isolation and Culture

The procedure for cell isolation and culture was performed as described previously [21]. Briefly, healthy human impacted third molars (*n* = 20) were gathered from sixteen young patients under the age of 20 in Oral Surgery Department of Jiangsu Provincial Stomatological Hospital. Root apical papilla was carefully isolated from the immature root apex. Primary apical papilla cells were enzymatically separated according to previous study [9] and cultured in alpha minimum essential medium (*α*-MEM, Gibco, Life Technologies, Grand Island, NY) supplemented with 10% fetal bovine serum (FBS, Hyclone, USA), 100 *μ*g/mL streptomycin, and 100 U/mL penicillin at 37°C in a humidified atmosphere of 5% CO_2_. Then, anti-rabbit IgG Dynabeads (Dynal Biotech, Oslo, Norway) and rabbit anti-STRO-1 antibody (Santa Cruz, Delaware, CA) were used to purify these isolated cells according to the standard operating procedures for magnetic activated cell sorting (MACS). TNF-*α* (Peprotech, USA) was dissolved in *α*-MEM at the concentration of 100 mg/mL and stored at −20°C. BMS-345541 (Sigma-Aldrich, MO) was dissolved in DMSO to produce a 50 *μ*mol/L stock solution. In consideration of the cellular cytotoxicity of chemical inhibitor at high concentration as revealed in previous study [15], we selected 1 *μ*mol/L BMS-345541 for the subsequent investigation.

### 2.2. Cell Identification

To determine the nature of cultured cells, isolated cells were immunostained with the antibody against STRO-1 (1 : 200, Novus Biologicals, USA) and cytokeratin (1 : 100, Bioworld, USA). Phosphate buffered saline (PBS) was simultaneously used as a control. Flow cytometric analysis of specific surface antigens was also used to characterize the cultured cells. Cells were harvested and incubated with various combinations of the following fluorochrome-conjugated rabbit anti-human antibodies: CD34-FITC, CD45-PerCP, CD90-PE, CD105-APC, CD146-APC, and CD73-PE (all from Miltenyi, Germany) for 20 min at room temperature in the dark. The corresponding mouse IgG isotype control antibodies conjugated to FITC, PE, APC, or PerCP were employed as negative controls in each experiment. Stained cells were washed twice with 0.01 mol/L PBS and analyzed using BD FACSCalibur (BD Biosciences, USA).

### 2.3. Proliferation and Migration Assay

The proliferation of SCAPs treated in NF-*κ*B-activated culture media containing 10 ng/mL TNF-*α* or NF-*κ*B-inhibited culture media containing 1 *μ*mol/L BMS-345541 was examined by 3-(4,5-dimethylthiazol-2-yl)-2,5-diphenyl-2,5-tetrazoliumbromide (MTT) assay and flow cytometry (FCM). SCAPs were seeded into 96-well plates (Nunc, Thermo Fisher Scientific Inc.) at an initial density of 2 × 10^3^ cells/well for 24 hours. At 60% confluence, cells were serum starved for 24 hours and treated with 10 ng/mL TNF-*α* or 1 *μ*mol/L NF-*κ*B inhibitor BMS-345541. After 0, 1, 3, 5, 7, and 9 days of coculture, MTT assay was performed according to the previous report [22]. The optical absorbance was obtained in an enzyme-linked immunosorbent assay plate reader (Titertek, Helsinki, Finland) at 490 nm according to the manufacturer's instruction. The data were presented as the means ± SD (*n* = 6) and this experiment was repeated in triplicate.

SCAPs (1 × 10^6^) in control, NF-*κ*B-activated, and NF-*κ*B-inhibited groups were, respectively, collected, washed twice with cold 0.01 mol/L PBS, and fixed in 70% ice-cold ethanol overnight at 4°C in the dark. DNA content analysis of these cells was carried out using FACScan flow cytometer (BD Biosciences, San Jose, CA). Then, cell cycle distributions (G_1_, S, and G_2_M phases) were described and compared. This experiment was repeated three times.

For the wound healing assay, SCAPs were cultured to 90% confluence in 100 mm culture dishes and then wounded by using a pipette tip to scratch the monolayers. The initial scratched areas were uniform across the different samples and permanently marked. Floating cells and debris were removed and cells were cultured in *α*-MEM supplemented with 10 ng/mL TNF-*α* or 1 *μ*mol/L BMS-345541. The marked areas in each group were photographed at 0, 6, 12, and 24 hours after the scratch with an inverted microscope.

### 2.4. Alkaline Phosphatase (ALP) Activity Assay and Alizarin Red Staining

For the evaluation of osteogenic differentiation, ALP activity was detected by using an ALP kit (Nanjing Jiancheng Technological Inc., Nanjing, China). Cells were plated at a density of 3 × 10^3^ cells per well into 96-well plates. Then the media were changed and cells were cultured in the complete media or the mineralization-inducing media (MM) containing *α*-MEM, 10% FBS, 100 *μ*g/mL streptomycin, 100 U/mL penicillin, 50 mg/L ascorbic acid, 2 mmol/L L-glutamine, 10 nmol/L dexamethasone, and 10 mmol/L *β*-glycerophosphate (Sigma). In addition, activator (TNF-*α*) or inhibitor (BMS-345541) was added into experiment groups. ALP activity was measured after 3, 5, and 7 days of culture. The relative ALP activity was normalized to the total protein content per sample.

After 2 weeks of mineralization induction, cells were fixed in ice-cold 70% ethanol for 30 minutes and stained with alizarin red (40 mM, pH = 4.2, Sigma-Aldrich) for 5 min at room temperature. Images were obtained using a scanner. Calcium contents were quantitatively analyzed according to our previous method [13]. The results were described as the means ± SD, and each experiment was performed for three times.

### 2.5. Real-Time Reverse Transcriptase-Polymerase Chain Reaction (Real-Time RT-PCR)

SCAPs were cultured in complete media or mineralization media (MM), supplementing activator or inhibitor to experiment groups. After 3 days or 7 days of incubation, total RNA was extracted from cells in each group using TRIzol reagent (Invitrogen, Carlsbad, CA) according to the manufacturer's instructions. Total RNA was subjected to reverse transcription with a PrimeScript RT Master Mix kit (TaKaRa, Dalian, China). The mRNA expressions of several osteoblastic/dentinogenic markers, including* ALP*, osteocalcin (*OCN*), bone sialoprotein (*BSP*), osterix (*OSX*), runt-related transcription factor 2 (*RUNX2*), osteopontin (*OPN*), dentin sialophosphoprotein (DSPP), and dentin matrix protein-1 (DMP-1), were quantified by real-time RT-PCR using SYBR Premix Ex Taq kit (TaKaRa Bio, Japan) and ABI 7300 real-time PCR system. Relative gene expression values were calculated by the 2^−ΔΔCt^ method as previously described [23].* GAPDH* (glyceraldehyde-3-phosphate dehydrogenase) was employed as reference housekeeping gene for normalizing mRNA levels. All PCR reactions were performed in triplicate and data were expressed as means ± SD. Primers used for real-time RT-PCR were as follows:* GAPDH*, 5′-GAAGGTGAAGGTCGGAGTC-3′ and 5′-GAGATGGTGATGGGATTTC-3′;* ALP*, 5′-GACCTCCTCGGAAGACACTC-3′ and 5′-TGAAGGGCTTCTTGTCTGTG-3′;* OCN*, 5′-AGCAAAGGTGCAGCCTTTGT-3′ and 5′-GCGCCTGGGTCTCTTCACT-3′;* BSP*, 5′-CTATGGAGAGGACGCCACGCCTGG-3′ and 5′-CATAGCCATCGTAGCCTTGTCCT-3′;* RUNX2*, 5′-TCTTAGAACAAATTCTGCCCTTT-3′ and 5′-TGCTTTGGTCTTGAAATCACA-3′;* OSX*, 5′-CCTCCTCAGCTCACCTTCTC-3′ and 5′-GTTGGGAGCCCAAATAGAAA-3′;* DSPP*, 5′-ATATTGAGGGCTGGAATGGGGA-3′ and 5′-TTTGTGGCTCCAGCATTGTCA-3′;* OPN*, 5′-CCAAGTAAGTCCAACGAAAG-3′ and 5′-GGTGATGTCCTCGTCTGTA-3′;* DMP-1*, 5′-CCCTTGGAGAGCAGTGAGTC-3′ and 5′-CTCCTTTTCCTGTGCTCCTG-3′.

### 2.6. Western Blot Analysis

Cultured cells in complete media or mineralization media (MM), with the presence of activator or inhibitor in experimental groups, were dissolved on ice for 20 min in RIPA lysis buffer (Beyotime, China) supplemented with 1 mM phenylmethylsulfonyl fluoride (PMSF, Beyotime). 20 *μ*g of protein from each sample was used for western blot analysis following the protocols in our previous study [14].

As for the detection of signaling pathway, SCAPs were cultured in serum-free media for 24 hours, followed by the treatment of 10 ng/mL TNF-*α* or 1 *μ*mol/L BMS-345541. At the indicated time points, the cytoplasm protein was extracted with a Keygen Kit (Keygen Biotech., China) and western blot was subsequently performed.

The primary antibodies in this experiment were as follows: OCN (1 : 1000, Millipore), BSP (1 : 1000, Abcam), OSX (1 : 1000, Abcam), RUNX2 (1 : 1000, Abcam), DSP (1 : 500, Santa Cruz), OPN (1 : 1000, Abcam), DMP-1 (1 : 1000, Novus), phosphor-P65 (1 : 1000, Cell Signaling), P65 (1 : 1000, Cell Signaling), phosphor-I*κ*B*α* (1 : 1000, Cell Signaling), I*κ*B*α* (1 : 1000, Cell Signaling), and *β*-ACTIN (1 : 1000, Bioworld). Target protein expression was then quantified according to the band intensity and standardized by the structure protein *β*-ACTIN with Image-Proplus 5.0 software. In brief, the integral optical density of the protein bands was calculated by using Image-Proplus 5.0 software. Then densitometry ratios between the target protein and *β*-ACTIN were obtained in a semiquantitative level and the histogram was then plotted. The experiment was performed for three times.

### 2.7. Statistical Analysis

Statistical analysis was performed by paired *t*-test and one-way analysis of variance (ANOVA). Follow-up comparisons between experiment groups and control group were then carried out using the Dunnet posttest analysis. Values of *P* < 0.05 were regarded as statistically significant.

## 3. Results

### 3.1. Identification of SCAPs

**Figure 1:**
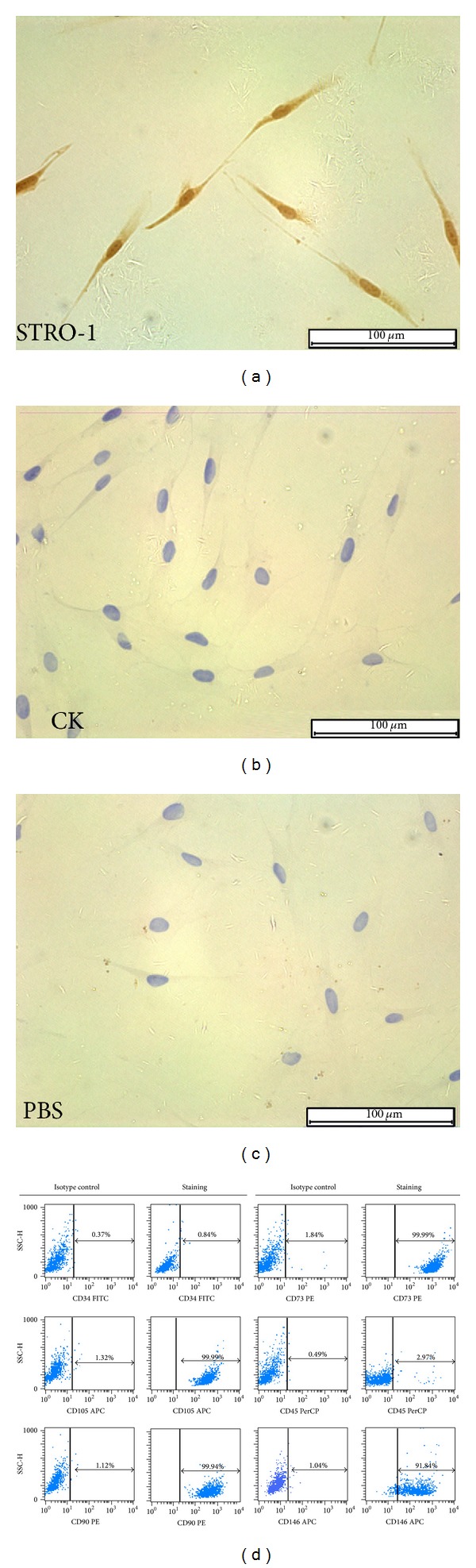
Characterization of SCAPs: (a) isolated SCAPs were positive for STRO-1 by immunocytochemistry; (b) isolated SCAPs were negative for CK by immunocytochemistry; (c) PBS served as a negative control; (d) flow cytometric analysis revealed that cultured SCAPs are positive for CD73 (99.99%), CD105 (99.99%), CD90 (99.94%), and CD146 (91.84%), but negative for CD34 (0.84%) and CD45 (2.97%). Mouse IgG isotype control antibodies conjugated to FITC, PE, APC, or PerCP were used as negative controls. Scale bars: 100 *μ*m.

Immunocytochemistry analysis showed that SCAPs were stained positively for the mesenchymal stem cell (MSC) surface molecule STRO-1 (Figure 1(a)), but negatively for epithelial cell marker cytokeratin (Figure 1(b)). Similarly, there was a high expression of MSC markers (e.g., CD29, CD73, CD90, CD105, and CD146), while the hematopoietic markers (e.g., CD34 and CD45) were low expressed in SCAPs as demonstrated by flow cytometry (Figure 1(d)). These data revealed the stromal origin of these isolated cells with stem cell characteristics and the absence of hematopoietic precursor contamination.

### 3.2. Activation and Inhibition of Canonical NF-*κ*B Signaling Pathway in SCAPs

**Figure 2:**
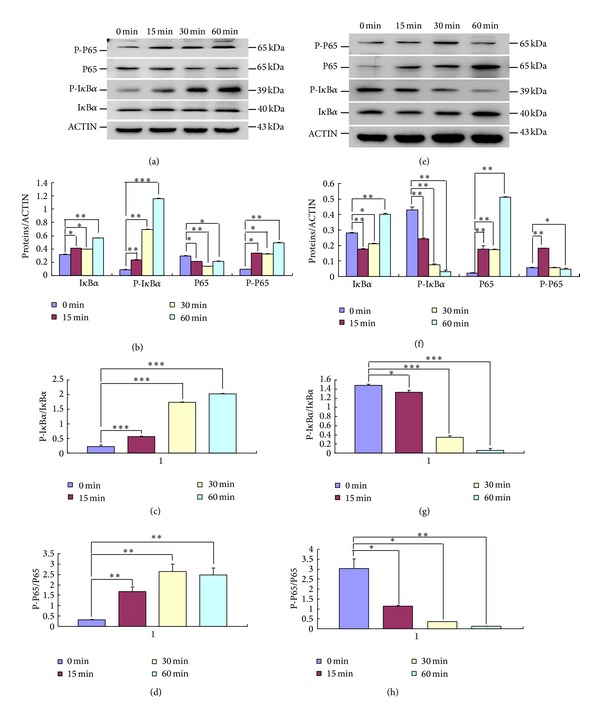
Activation and inhibition of canonical NF-*κ*B signaling pathway in SCAPs. (a) The expression of NF-*κ*B pathway proteins in TNF-*α*-treated SCAPs at different time points. (b) Semiquantitative analysis confirmed the upregulation of P-I*κ*B*α* and P-P65 after the activation of NF-*κ*B pathway. (c) The ratio of phosphorylated to unphosphorylated form of P65. (d) The ratio of phosphorylated I*κ*B*α* to unphosphorylated I*κ*B*α*. (e) The protein levels of I*κ*B*α*, P-I*κ*B*α*, P65, and P-P65 in the cytoplasm of BMS-345541-treated SCAPs at indicated time points. (f) Semiquantitative analysis confirmed the declined expression of P-I*κ*B*α* and P-P65 after the inhibition of NF-*κ*B pathway. (g) The ratio of phosphorylated to unphosphorylated form of P65. (h) The ratio of P-I*κ*B*α* to I*κ*B*α*. Values are the means ± SD; *n* = 3; **P < 0.01; ***P < 0.001.

It has been extensively proven that TNF-*α* is a potent activator of canonical NF-*κ*B pathway, while BMS-345541 is the highly selective inhibitor of NF-*κ*B. To determine whether SCAPs treated with TNF-*α* or BMS-345541 can result in the NF-*κ*B activation or inhibition, respectively, cytoplasm protein was extracted and subjected to electrophoresis. In TNF-*α*-treated SCAPs, phospho-I*κ*B*α* was obviously elevated in a time-dependent manner and phosphorylated P65 rapidly reached a maximal increase within 15 minutes after TNF-*α* stimulation (Figures 2(a) and 2(b)). Suppressed NF-*κ*B activity was detected in SCAPs after incubation with BMS-345541, as indicated by the decreased phosphorylated I*κ*B*α* and P65 (Figures 2(e) and 2(f)). Ratios of phosphorylated to unphosphorylated forms of proteins further confirmed the activation of NF-*κ*B by TNF-*α* and inhibition of NF-*κ*B by BMS-345541 (Figures 2(c), 2(d), 2(g), and 2(h); *P* < 0.05).

### 3.3. Effects of Canonical NF-*κ*B Pathway on the Proliferation of SCAPs

**Figure 3:**
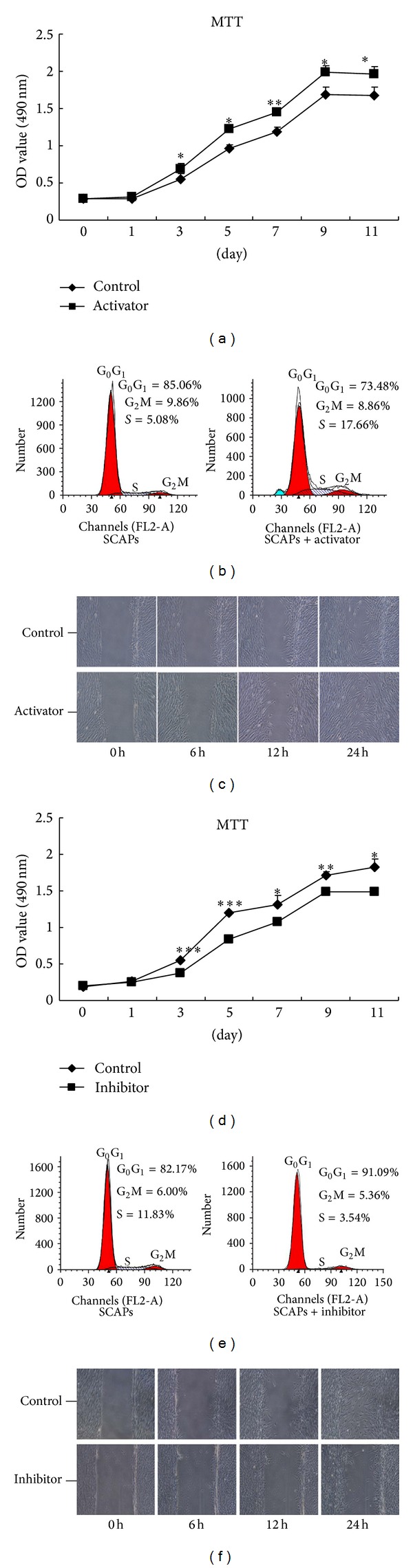
Effects of canonical NF-*κ*B signaling pathway on the proliferation of SCAPs. (a) Cell proliferation in NF-*κ*B pathway-activated and control groups was detected by MTT assay. (b) Flow cytometry analysis for SCAPs in NF-*κ*B pathway-activated and untreated groups. The proliferation index (PI = S% + G_2_M%) in NF-*κ*B pathway-activated group (26.52%) was significantly higher than that in control group (14.94%). (c) Images of scratch wounds in NF-*κ*B pathway-activated and untreated groups at indicated time points. (d) Growth curves of NF-*κ*B pathway-inhibited and untreated SCAPs. (e) Representative cell cycle distributions of NF-*κ*B pathway-inhibited and untreated SCAP. The proliferation index (PI = S% + G_2_M%) in NF-*κ*B pathway-activated group (8.90%) was obviously lower than that in control group (17.83%). (f) Cell motility of SCAPs in NF-*κ*B pathway-inhibited and untreated group assessed by a scratch assay. Values were the means ± SD; *n* = 6; *P < 0.05; **P < 0.01; ***P < 0.001. Scale bars = 100 *μ*m.

As shown in Figure 3(a), SCAPs in activator group exhibited higher proliferation, while the SCAPs-inhibitor group showed less proliferation capacity as compared with the corresponding control groups, respectively (*P* < 0.05), except for the time points at baseline (day 0) and the first day. Flow cytometry assay revealed that the activator-treated SCAPs exhibited a higher percentage of cells in S and G_2_M phases (26.52%) and a lower percentage of cells in G_0_G_1_ phase (73.48%) in comparison with untreated cells (*P* < 0.05; Figure 3(b)). There was a lower percentage (8.90%) of proliferating cells in S/G_2_M phases in the inhibitor-treated SCAPs as compared with the control group (17.83%) at day 3 (Figure 3(e)), which is consistent with the findings in MTT assay (Figure 3(d)). These results indicate that canonical NF-*κ*B pathway facilitated the proliferation of SCAPs, that is, the cell proliferation rate increasing in pace with the activation of canonical NF-*κ*B, and is restrained along with the blockage of NF-*κ*B. Furthermore, an improved migration ability was observed in activator-treated SCAPs (Figure 3(c)), while scratch closure was markedly slower in SCAPs treated with inhibitor (Figure 3(f)).

### 3.4. Effects of Canonical NF-*κ*B Pathway on the Odonto/Osteogenic Differentiation of SCAPs

**Figure 4:**
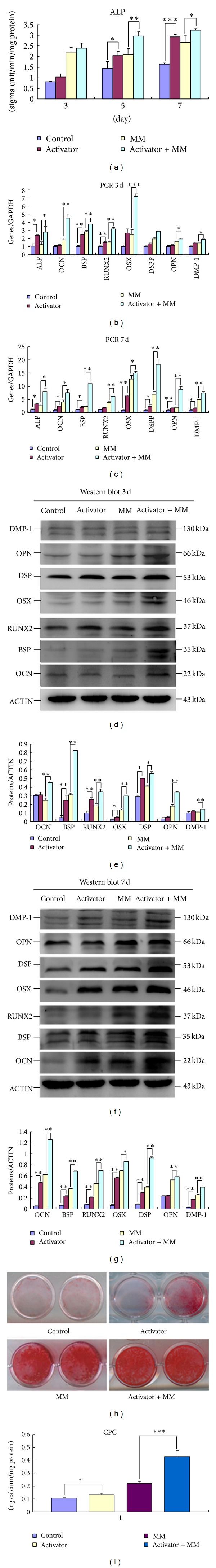
Odonto/osteogenic differentiation in canonical NF-*κ*B-activated SCAPs. (a) ALP activity of SCAPs in control, activator, MM, and activator + MM groups at days 3, 5, and 7. Values are the means ± SD; *n* = 6; *P < 0.05; **P < 0.01; ***P < 0.001. (b) Real-time RT-PCR analysis for odonto/osteogenic genes (*BSP*,* OCN*,* RUNX2*,* OSX*,* DSP*,* OPN*,* DSP*, and* DMP-1*) in each group at day 7. (c) Real-time RT-PCR analysis for odonto/osteogenic genes (*ALP*,* BSP*,* OCN*,* RUNX2*,* OSX*,* DSP*,* OPN*,* DSP*, and* DMP-1*) in different groups at day 7.* GAPDH* served as a housekeeping gene. **2^−ΔΔCt^ ≥ 2, *P* < 0.01; *1 < 2^−ΔΔCt^ < 2, *P* < 0.01; *n* = 3. (d) Western blot analyses for the odonto/osteogenic proteins (BSP, OCN, RUNX2, OSX, DSP, OPN, DSP, and DMP-1) in different groups at day 3. *β*-ACTIN served as an internal control. (e) Semiquantitative analysis demonstrated that the expression of BSP, OCN, RUNX2, OSX, DSP, OPN, DSP, and DMP-1 was stronger in NF-*κ*B pathway-activated SCAPs than those in control group at day 3, especially in the presence of mineralization-inducing media. (f) Western blot analyses for the odonto/osteogenic proteins (BSP, OCN, RUNX2, OSX, DSP, OPN, DSP, and DMP-1) in different groups at day 7. *β*-ACTIN was used as an internal control. (g) Semiquantitative analysis confirmed that the expression of BSP, OCN, RUNX2, OSX, DSP, OPN, DSP, and DMP-1 was upregulated in NF-*κ*B pathway-activated SCAPs at day 7, regardless of the presence or absence of mineralization-inducing media. (h) Alizarin red staining of SCAPs after 14 days of induction in different groups. (i) Quantitative calcium analysis showed that the calcium content in activator and activator + MM groups was significantly higher than the control and MM groups, respectively. Values were described as the means ± SD. *P < 0.05; ***P < 0.001.

ALP activities in activator-treated SCAPs in basic medium and mineralization-inducing medium were elevated at day 5 and day 7 (*P* < 0.05, Figure 4(a)), as compared with untreated groups. The density of calcification nodules was significantly higher in activator-stimulated groups than in the other groups after 14 days of coculture (Figure 4(h)). Moreover, quantitative calcium measurement illustrated more calcifications in activator-treated SCAPs in comparison with untreated groups (Figure 4(i)).

Differentially expression levels of related osteo/odontogenic genes were also investigated by real-time RT-PCR assays. In activator-treated group, expression of specific osteo/odontogenic genes (e.g.,* ALP*,* OCN*,* BSP*,* OSX*,* RUNX2*,* DSP*,* OPN*, and* DMP-1*) was significantly upregulated at days 3 and 7 (Figures 4(b) and 4(c)). At day 3, the expression of some osteo/odontogenic genes in activator-treated groups did not change obviously in complete media but was distinctly increased in the presence of mineralization-inducing media. At day 7, the odonto/osteogenic markers were significantly upregulated following the activator treatment regardless of the presence or absence of mineralization-inducing media. This phenomenon was not observed in NF-*κ*B-inhibition group. These findings were confirmed by western blot assay in which activated classical NF-*κ*B signaling pathway induced a significant increase of related protein expression (Figures 4(d)–4(g)).

**Figure 5:**
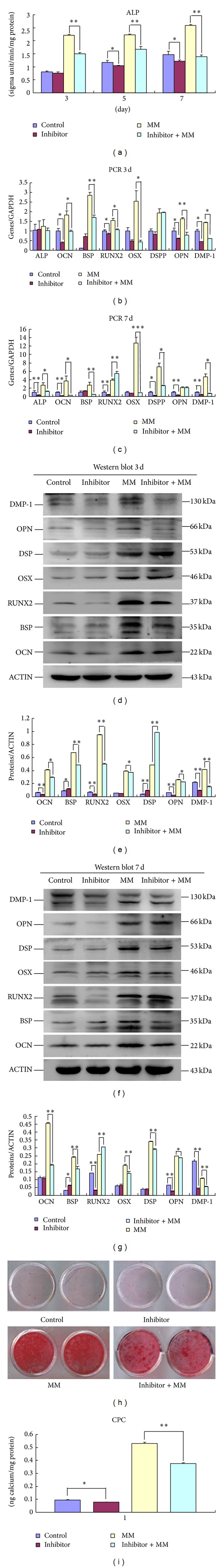
Odonto/osteogenic differentiation in canonical NF-*κ*B-inhibited SCAPs. (a) ALP activity at different time points in different groups (i.e., control, inhibitor, MM, and inhibitor + MM). (b) Real-time RT-PCR for the detection of* ALP*,* BSP*,* OCN*,* RUNX2*,* OSX*,* DSP*,* OPN*,* DSP*, and* DMP-1* at day 3. The mRNA levels were normalized to* GAPDH*. (c) Real-time RT-PCR for the detection of* ALP*,* BSP*,* OCN*,* RUNX2*,* OSX*,* DSP*,* OPN*,* DSP*, and* DMP-1* of SCAPs, respectively, in control, inhibitor, MM, and inhibitor + MM groups at day 7. (d) The odonto/osteogenic protein levels of SCAPs in control, inhibitor, MM, and inhibitor + MM groups were characterized by western blot analysis at day 3. *β*-ACTIN was used as a loading control. (e) Semiquantitative analysis demonstrated that the expression of BSP, OCN, RUNX2, OSX, DSP, OPN, DSP, and DMP-1 was more downregulated in NF-*κ*B pathway-inhibited SCAPs than those in control group at day 3. (f) The odonto/osteogenic protein levels of SCAPs in control, inhibitor, MM, and inhibitor + MM groups were characterized by western blot analysis at day 7. (g) Semiquantitative analysis demonstrated that the expression of odonto/osteogenic markers (BSP, OCN, RUNX2, OSX, DSP, OPN, DSP, and DMP-1) was significantly more downregulated in NF-*κ*B pathway-inhibited SCAPs than those in control group at day 7. (h) Alizarin red staining of SCAPs after 14 days of osteogenic induction. (i) Calcium quantification demonstrated the weaker calcium deposition in inhibitor and inhibitor + MM groups, as compared with control and MM groups, respectively.

As shown in Figure 5(a), the blockage of NF-*κ*B signaling pathway obviously downregulated the ALP activity at days 3, 5, and 7 (*P* < 0.05). At day 14, less calcified nodules were generated in inhibitor-treated groups (Figure 5(h)). Calcium quantification also revealed the less calcium deposition in inhibitor and inhibitor + MM groups, as compared with control and MM groups, respectively (Figure 5(i), *P* < 0.05). There was a remarkable decrease of osteo/odontogenic genes at different time points (Figures 5(b) and 5(c)). Western blot analysis further verified these findings (Figures 5(d)–5(g)).

## 4. Discussion

SCAPs are known as a kind of ideal candidates for dental tissue engineering and have the characteristics of self-renewal and multilineage differentiation potential [9, 10]. Diverse studies have proved that SCAPs are able to differentiate into osteo/odontoblasts* in vitro* under appropriate conditions and form bone/dentin-like tissues* in vivo* [24, 25]. Certainly, many signaling pathways may be involved in the process of cell proliferation and differentiation including NF-*κ*B pathway.

NF-*κ*B exists in the cytoplasm in a latent form binding to inhibitory proteins termed inhibitory *κ*B proteins (I*κ*Bs) [3, 26]. I*κ*B complex is the major regulator of NF-*κ*B pathway and is composed of catalytic subunits IKK*α* and IKK*β* and a regulatory subunit IKK*γ*. TNF-*α* is generally known to activate classical NF-*κ*B pathway [17]. Exposure of cells to suitable concentrations of TNF-*α* brings about the rapid phosphorylation, ubiquitination, and proteolytic degradation of I*κ*B, which allows NF-*κ*B to translocate to the nucleus, where it subsequently regulates the gene transcription [27–29]. BMS-345541, a selective inhibitor of the catalytic subunits of IKK, targets NF-*κ*B signaling pathway and downregulates the activity of NF-*κ*B [18, 19]. In this study, the expression of cytoplastic P-P65/P-I*κ*B*α* was noticeably upregulated after the treatment of TNF-*α* and downregulated by the inhibitor BMS-345541, indicating the successful establishment of a cellular model for the activation or suppression of canonical NF-*κ*B pathway.

Cell proliferation and migration were indispensable for tissue development and wound healing. In the present study, NF-*κ*B-activated SCAPs exhibited an increased growth rate, higher proliferation index, and more effective migration in wound healing assay. Moreover, the migration distances between the wound lines in inhibitor-treated cells were longer than control cells after 24 hours of culture. Together with the weakened proliferation of inhibitor-treated cells, it is predictable that the canonical NF-*κ*B pathway has a positive influence on the cell multiplication activity and motility.

Previous studies have proved that various stimuli can exert an active role on the committed differentiation of dental stem cells via NF-*κ*B pathway [28]. In the present study, the odonto/osteogenic capacity remarkably changed along with the activation or blockage of classical NF-*κ*B pathway. Activator-treated SCAPs exhibited an enhanced ALP activity, increased calcium deposition, and upregulated expression of odonto/osteogenic genes and proteins, while inhibitor-treated cells presented the lower ALP activity, decreased mineralization, and downregulated expression of odonto/osteoblast markers.

It is widely recognized that ALP is a functional marker for osteoblast activity and bone formation [30]. As an important extracellular matrix of bone, BSP is mainly secreted by osteoblasts whose expression is a crucial symbol in matrix deposition and mineralization [31, 32]. OPN is another extracellular matrix protein expressed in numerous cell types including odontoblasts, in which OPN plays multifaceted roles in a variety of biological and pathological processes, such as osteogenic differentiation, tooth mineralization, and dental biofilm formation [33, 34]. OCN is the primary noncollagenous protein in the bone cells secreted in the late stage of osteogenesis and known as a specific indicator of osteogenic differentiation. RUNX2 is the first transcription factor required for determination of the osteoblast lineage and OSX acts as a downstream gene of RUNX2 that is highly expressed in the functional odonto/osteoblasts [35].* DSPP* and DSP are well-known markers of odontoblasts, highly expressed in dentin or predentin structures and essential for dentinogenesis [36]. DMP-1 is an acidic extracellular matrix protein that is primarily found in dentin and bone and has been implicated in dentin mineralization and signal transduction in the process of odontogenesis [37].

In this study, the expression of* RUNX2*/RUNX2 reduced after 3 days of incubation with the inhibitor BMS-345541 and then gradually increased at day 7, while the expression of* DSPP*/DSP did not compromise after 3 days of incubation but noticeably decreased at day 7, indicating that the expression of* RUNX2*/RUNX2 and* DSPP*/DSP tends to be inversely correlated [38]. Moreover, the dramatic difference of these odonto/osteogenic markers at both mRNA and protein levels in response to the regulation of NF-*κ*B pathway suggests that activated canonical NF-*κ*B pathway favors the odonto/osteoblastic differentiation of SCAPs, particularly in the presence of mineralization-inducing media.

In summary, the findings accumulated here confirmed the potential involvement of canonical NF-*κ*B pathway in the proliferation, migration, and committed differentiation of SCAPs* in vitro*. Both activation and inhibition of the classical NF-*κ*B pathway can bring about the permanent changes in human stem cells. Moreover, it is commonly believed that NF-*κ*B signaling plays a pivotal role not only in the progress of normal physiological process but also in the pathological process, and dysfunction of NF-*κ*B is linked to various human diseases. Thus, proper balance of intracellular NF-*κ*B should be maintained in the physiological conditions, while intricate interactivity between NF-*κ*B and other signaling pathways needs to be extensively investigated.
